# All organic homojunction PEDOT:PSS p–n diode

**DOI:** 10.1038/s41598-022-16432-8

**Published:** 2022-07-21

**Authors:** S. Aboulhadeed, M. Ghali, M. M. Ayad

**Affiliations:** 1 Department of Physics, Institute of Basic and Applied Science, Egypt-Japan University of Science and Technology, Alexandria, Egypt; 2 Department of Chemistry, Institute of Basic and Applied Science, Egypt-Japan University of Science and Technology, Alexandria, Egypt; 3 Polymer Materials Research Department, Advanced Technologies and New Materials Research Institute, City of Scientific Research and Technological Applications, Alexandria, Egypt; 4 Physics Department, Kafrelsheikh University, Kafrelsheikh, Egypt; 5 Chemistry Department, Tanta University, Tanta, Egypt

**Keywords:** Energy science and technology, Materials science

## Abstract

It is widely known that poly(3,4-ethylene dioxythiophene)-poly(styrenesulfonate) (PEDOT:PSS) is only a p-type material, and thus there is a challenge to fabricating all PEDOT:PSS based p–n device. Here, and for the first time, we introduce a new homojunction p–n diode device based solely on PEDOT:PSS thin films. The diode shows a nonlinear I–V behavior with a rectification ratio of 3 and a turn-on voltage ~ 1.4 V.

## Introduction

Conductive Poly(3,4-ethylene dioxythiophene) poly(styrene sulfonate) polymer (PEDOT:PSS) is typically utilized as a p-type hole transport material in polymer solar cells and *heterojunction* Schottky diodes due to its high optical transparency, particularly in the visible light^1,2^. However, the electrical conductivity of pristine p-PEDOT:PSS requires enhancement because of its natural core–shell structure. It is widely known that the grains of PEDOT:PSS are typically surrounded by the electrically insulating PSS^3^. Previous works discussed possible enhancement of p-PEDOT:PSS conductivity using, e.g., organic solvents^4^. Very few works reported on the development of n-type PEDOT:PSS films^5–7^, even with limited success in utilizing n-PEDOT:PSS in efficient homojunction devices like a simple p–n diode. For example, Lu et al., utilized isopropanol-treated PEDOT:PSS as n-type layer in a heterojunction device with p-(P3HT: PCBM) BHJ-PSC material^8^. Also, Chen et al., obtained n-PEDOT:PSS by spin coating PEIE solvent over p-PEDOT:PSS layer^9^. On the other hand, Kim et al., converted p-PEDOT:PSS film into n-type to function as thermoelectric materials by simply treating p-PEDOT:PSS with 40% wt of CuCl_2_^10^.

Also Sami et al., made a thin film solar cell based on n-PEDOT:PSS/p-CuInGaSe_2_ heterostructure. Yet, all these works did not give information on the physical properties of the resultant n-PEDOT:PSS layer or the reasons behind PEDOT:PSS conversion from p to n-type or possibility to fabricate all PEDOT:PSS homojunction device. Here, we report on details of p-PEDOT:PSS to n-PEDOT:PSS conversion process where isopropanol solvent was utilized for this conversion and found to be highly influential on the physical properties of the resultant n-PEDOT:PSS layer. Importantly, the developed n-PEDOT:PSS was used in a new *homojunction* diode structure of FTO/p-PEDOT:PSS/n-PEDOT:PSS/Cu where p and n-type PEDOT thin layers were solely used.

## Experimental details

PEDOT:PSS with a concentration of 1.1% in H_2_O (model number: 739324) and isopropanol alcohol (ISO) (99.5%, extra dry) were obtained from Sigma-Aldrich and used directly without any further purification. PEDOT:PSS thin films were prepared as follows: A 1 mL of PEDOT:PSS solution was blended with 1 mL of ISO (v:v) under stirring at 55 °C. After that, the resultant mixture (i.e., PEDOT:PSS/ISO) was deposited onto a cleaned glass substrate (1 cm^2^ × 1 cm^2^) at 120 °C for 2 min using a film applicator with a speed of 7 mm/s (model COATMASTER 510XL). The obtained PEDOT:PSS/ISO film was annealed and cured for 10 min at 120 °C. To fabricate the PEDOT:PSS homojunction diode, the structure of FTO/p-PEDOT:PSS/n-PEDOT:PSS/Cu was made by: (1) depositing a 20 μL of pristine PEDOT:PSS solution on FTO substrate at 120˚C. Then, the film was left to dry under N_2_ atmosphere for 5 min; lastly, the obtained film was annealed at 120˚C in a vacuum oven for 10 min; (2) a 20 μL of PEDOT:PSS solution, blended with ISO in a volume ratio (1:1) (v:v), was deposited on the p-pristine p-PEDOT:PSS layer using the same procedure of drying and annealing. Finally, an ohmic contact of ~ 50 nm thick Cu metal was thermally evaporated and deposited on the n-PEDOT:PSS layer as a top electrode.

## Results and discussion

**Figure 1:**
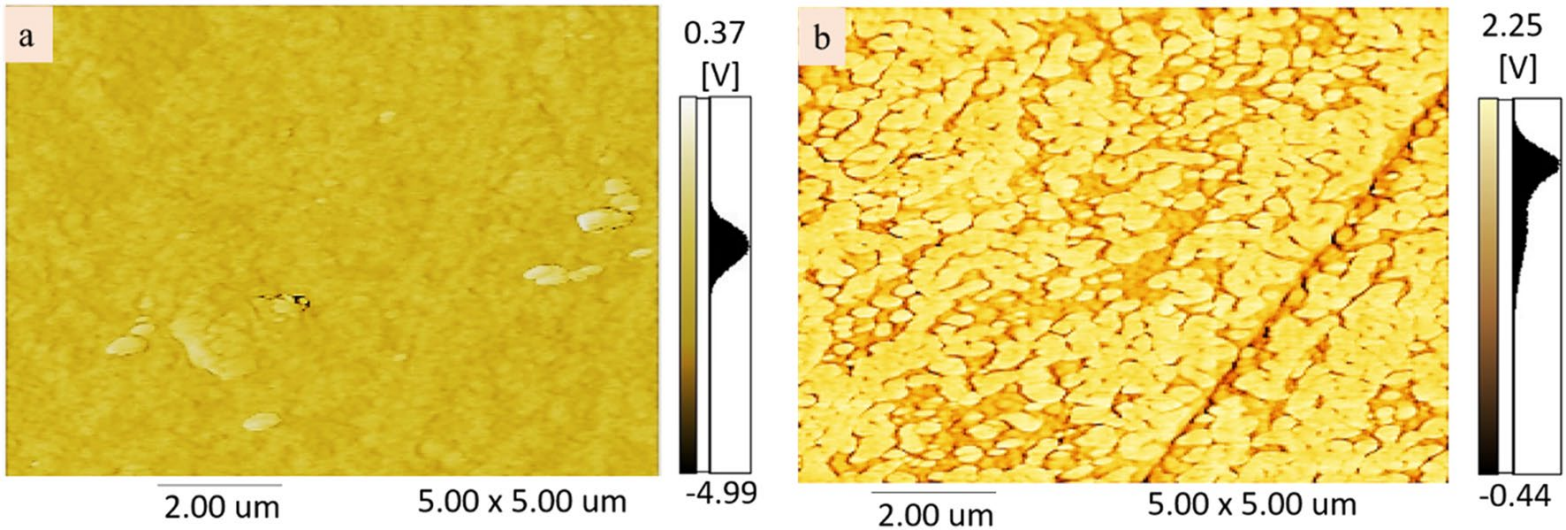
AFM phase images of PEDOT:PSS films without (**a**) and with isopropanol treatment (**b**). All images are 5 µm × 5 µm.

Figure 1 shows Atomic Force Microscope (AFM) measurements where the phase images of pristine and PEDOT:PSS/ISO films are compared. For the pristine PEDOT:PSS film, Fig. 1a, it is noticed that the phase image demonstrates clearly a weak phase separation between the PEDOT and the PSS chains. In contrary, for the n-type PEDOT:PSS/ISO, Fig. 1b, the phase image shows a strong phase separation between the PEDOT and the PSS chains. Furthermore, the AFM images in Fig. 1b prove a much larger grain size and much more interconnected PEDOT chains in the case of the n-PEDOT:PSS film. Also, the n-PEDOT:PSS/ISO film shows a surface roughness of 5.3 nm compared to 14.8 nm for the pristine p-PEDOT:PSS film. We ascribe the improvement of the film surface roughness to the observed reduction and the separation of PSS from PEDOT in the structure. It is most probable that the OH group of isopropanol controls the role of PSS and thus reduces the hydrophobic character of PEDOT. These AFM images confirm that isopropanol plays a significant role in the phase separation process between the conductive PEDOT and the insulating PSS within the PEDOT:PSS^11–13^. In addition, and due to the hydrophilic properties of isopropanol, the phase-separated PSS chains are most likely to be dissolved and thus removed from the PEDOT:PSS film surface.

**Figure 2:**
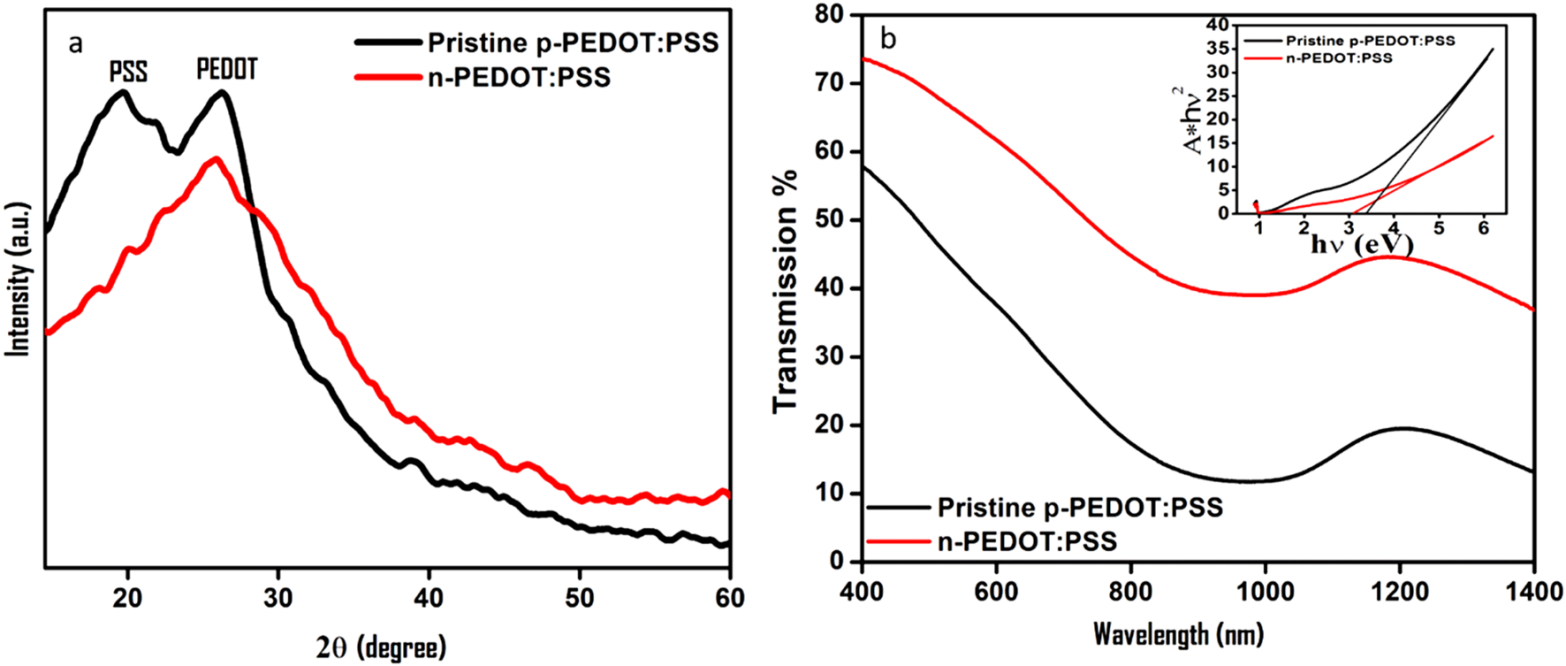
(**a**) XRD pattern and (**b**) transmittance spectra of pristine and PEDOT:PSS/ISO films. The insets show the estimated bandgap.

Figure 2a shows the X-ray diffraction measurement (XRD) of the deposited pristine p-PEDOT:PSS and the n-type PEDOT:PSS/ISO (1:1) films. XRD of the pristine p-PEDOT:PSS shows broad two prominent peaks at 2θ = 19.4° and 2θ = 26°, which are attributed to the PSS and the inner chain-ring stacking distance PEDOT with the crystallographic plane (020)^14^, respectively.

For the n-PEDOT:PSS/ISO (1:1), it is clear that the PSS-related XRD peak is declined, which confirms a significant separation between the PSS and PEDOT due to interaction with isopropanol and/or dissolving of the phase-separated PSS chain in isopropanol. Optical transmission measurements of the pristine p-PEDOT:PSS and n-PEDOT:PSS films were conducted using a UV–Vis-NIR spectrophotometer (Hitachi model UH 5700) (Fig. 2b). It is noticed that the transmittance of the n-PEDOT:PSS/ISO film has been improved compared with that of the pristine p-PEDOT:PSS. Such enhancement can be ascribed to better alignment of the polymer chains upon interaction with the isopropanol where the chains take a quinoid structure. While for the p-PEDOT:PSS, the defects and disorders along the benzoid structure polymeric chain limit the carrier-free path. Besides, film roughness plays a role in the optical properties of films where n-PEDOT:PSS film displays a much lower surface roughness compared to that for p-PEDOT:PSS film. Optical bandgap (*E*_*g*_) of PEDOT:PSS films was calculated by plotting (Ahν)^2^ versus (hν), where “*A*” is the optical absorbance, “*h*” Planck's constant, “ν” is the frequency. The linear portion of the (Ahν)^2^–(hν) plot was extrapolated in the bandgap region, and the *E*_*g*_ was determined to be 3.38 eV and 3.1 eV for the p-PEDOT:PSS and the n-PEDOT:PSS films, respectively (Fig. 2b, inset).

**Figure 3:**
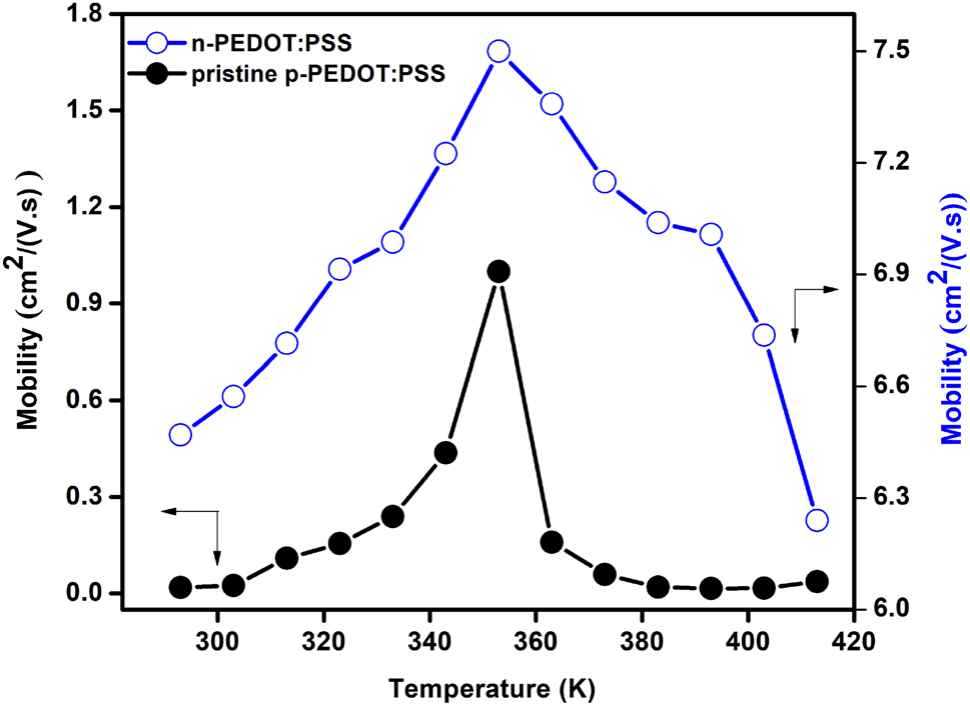
Temperature dependence of the mobility of pristine p-PEDOT:PSS and n-PEDOT:PSS films.

**Figure 4:**
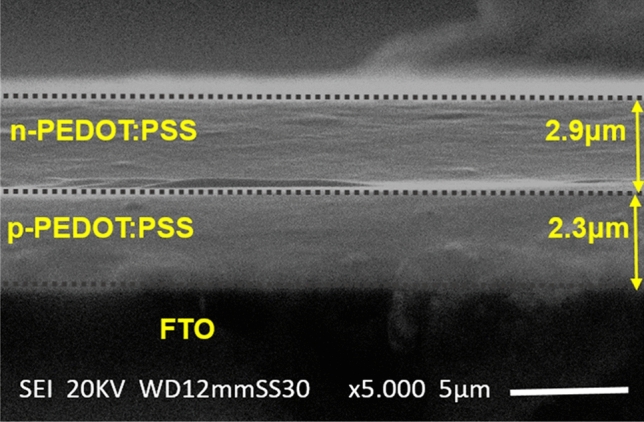
SEM cross-sectional image of the fabricated PEDOT:PSS homojunction p–n diode (p-PEDOT:PSS/n-PEDOT:PSS).

Hall-effect measurements were conducted where information on the mobility µ, resistivity ρ, carrier concentration n, conductivity σ, and Hall coefficient RH information was collected. Ecopia Hall-effect system (model: HMS-3000) was used where a magnetic field strength B = 0.53 T was utilized for the measurements. Room temperature Hall-effect results are summarized in Table 1. As seen, the PEDOT:PSS/ISO film shows n-type character with substantial increase of the mobility and conductivity compared with that of pristine p-PEDOT:PSS film which can be ascribed to the separation between the PSS and PEDOT and dissolving of PSS chain upon interaction with isopropanol. Moreover, dependence of the charge carrier mobility on temperature is measured and the data are shown in Fig. 3. We notice that mobility increases with temperature, indicating semiconductor behavior, until it reaches a maximum value at ~ 350 K. Such increase in the mobility can be ascribed to better alignment of chains in the PEDOT:PSS structure due to thermal heating^15^. Further increase in temperature leads to phonon interactions which results in an increase in electron scattering and thereby reduces the mobility. We utilized the developed n-PEDOT:PSS/ISO film in a new p–n diode structure device. Experimentally, we fabricated FTO/p-PEDOT:PSS/n-PEDOT:PSS/Cu device by firstly drop-casting ~ 2.3 µm thick layer of pristine p-PEDOT:PSS ink on FTO-coated glass substrate. Then, a ~ 2.9 µm thick layer of n-PEDOT:PSS was consequently also drop-casted onto the deposited p-PEDOT:PSS film at 120 °C for 10 min. Figure 4 shows the experimental cross-sectional SEM image of the FTO/p-PEDOT:PSS/n-PEDOT:PSS device. The image proves that the p-PEDOT:PSS layer spreads smoothly on the FTO substrate with no overlapping or dislocations at the p–n interface. A Cu metal was used as a top electrode while the FTO substrate was utilized as the bottom contact. We examined the I–V curve of the Cu electrode and the n-PEDOT:PSS layer to validate that the diode behavior is correlated only to the prepared homojunction based PEDOT:PSS diode.

**Table 1:** Room temperature Hall-effect data of pristine and isopropanol-treated PEDOT:PSS (1:1) (v:v) films.

	Mobility µ (cm^2^/Vs)	Resistivity ρ (Ω cm)	Carrier Concentrations (1/cm^3^)	Conductivity σ (1/Ω cm)	Hall coefficient RH (cm^3^/C)
Pristine PEDOT:PSS	0.02	0.31	8.38E20	2.95	0.86
PEDOT:PSS/ISO (1:1) (v:v)	6.57	0.09	− 5.59E21	13.25	− 0.50

**Figure 5:**
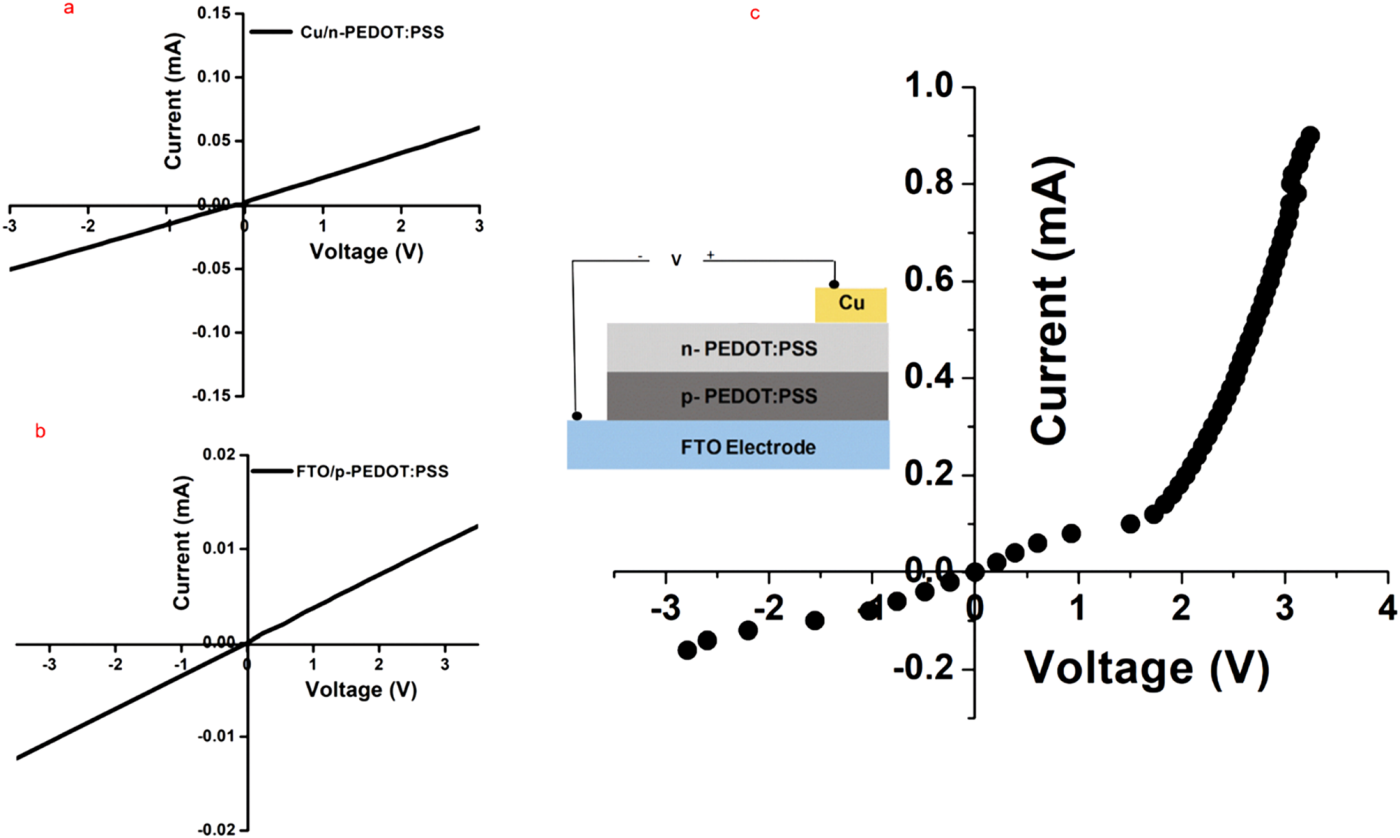
(**a**). I–V curve of (**a**) Cu electrode and n-PEDOT:PSS and (**b**) FTO and p-PEDOT:PSS layers. (**c**) I–V curve of FTO/p-PEDOT:PSS/n-PEDOT:PSS/Cu homojunction diode with area of 0.5 cm^2^. The inset is schematic structure of the device.

As seen in Fig. 5a, the measured I–V curve show a linear relation and thus confirms an ohmic contact between the Cu electrode and the n-PEDOT:PSS layer. A similar behavior was found for the FTO and the p-PEDOT:PSS (Fig. 5b). The device performance was studied using (I–V) measurements. Figure 5c presents the obtained I–V curve where the device parameters are extracted. The device shows a reasonable rectification factor of 3. Although such rectification ratio is lower than that of typical inorganic heterojunction diodes (e.g., SnO_2_/PEDOT:PSS/PVP and PANI:PEDOT:PSS blends)^16,17^, yet it is still higher than that of organic/inorganic heterojunction diodes (e.g., PEDOT:PSS-PVA/n-Si, and Al/5,14-dihydro-5,7,12,14-tetraazapentacenes (DHTAPs))^18,19^. For improvement of the rectification ratio, one can consider: (1) improving the diode ideality factor via reducing the non-homogeneity in the PEDOT:PSS film thickness and the irregularities of the barrier height by optimizing the thin film deposition technique; (2) enhancing the carriers injection efficiency via e.g., diminish the energetic difference between the electrode work-function and the PEDOT:PSS highest-occupied molecular orbital; (3) engineering the p-PEDOT:PSS/n-PEDOT:PSS interface for more efficient electron and hole transport.

## Conclusion

A simple homojunction nonlinear organic p–n diode device based on PEDOT:PSS films was fabricated and proved rectification ratio of 3. These results may stimulate new research on organic/organic or organic/inorganic electronic devices where n-PEDOT:PSS/ISO can play a novel role^20–23^.

## Data Availability

The data presented in this study are available on request from the corresponding author.
